# Impact of root-associated strains of three *Paraburkholderia* species on primary and secondary metabolism of *Brassica oleracea*

**DOI:** 10.1038/s41598-021-82238-9

**Published:** 2021-02-02

**Authors:** Je-Seung Jeon, Natalia Carreno-Quintero, Henriëtte D. L. M. van Eekelen, Ric C. H. De Vos, Jos M. Raaijmakers, Desalegn W. Etalo

**Affiliations:** 1 Department of Microbial Ecology, Netherlands Institute of Ecology NIOO-KNAW, Wageningen, 6708 PB The Netherlands; 2 Institute of Biology, Leiden University, Leiden, 2333 BE The Netherlands; 3 KeyGene N.V., Wageningen, 6708 PW The Netherlands; 4 Wageningen Plant Research, Bioscience, Wageningen, 6708 PB The Netherlands

**Keywords:** Biochemistry, Chemical biology, Ecology, Microbiology, Plant sciences, Systems biology

## Abstract

Several root-colonizing bacterial species can simultaneously promote plant growth and induce systemic resistance. How these rhizobacteria modulate plant metabolism to accommodate the carbon and energy demand from these two competing processes is largely unknown. Here, we show that strains of three *Paraburkholderia* species, *P. graminis* PHS1 (*Pbg*), *P. hospita* mHSR1 (*Pbh*), and *P. terricola* mHS1 (*Pbt*), upon colonization of the roots of two Broccoli cultivars led to cultivar-dependent increases in biomass, changes in primary and secondary metabolism and induced resistance against the bacterial leaf pathogen *Xanthomonas campestris.* Strains that promoted growth led to greater accumulation of soluble sugars in the shoot and particularly fructose levels showed an increase of up to 280-fold relative to the non-treated control plants. Similarly, a number of secondary metabolites constituting chemical and structural defense, including flavonoids, hydroxycinnamates, stilbenoids, coumarins and lignins, showed greater accumulation while other resource-competing metabolite pathways were depleted. High soluble sugar generation, efficient sugar utilization, and suppression or remobilization of resource-competing metabolites potentially contributed to curb the tradeoff between the carbon and energy demanding processes induced by *Paraburkholderia*-Broccoli interaction. Collectively, our results provide a comprehensive and integrated view of the temporal changes in plant metabolome associated with rhizobacteria-mediated plant growth promotion and induced resistance.

## Introduction

Plants allocate their photoassimilates for growth, storage, and defense, but they also release them into the rhizosphere feeding the microbial community^1,2^. Particularly, root exudation of soluble organic carbon is often omitted from the plant carbon balance as it is considered a minor component^3^. However, several studies suggested that 20–50% of the total fixed carbon can be released belowground from plant roots^4–6^. Low molecular weight organic compounds including amino acids, organic acids, sugars, phenolics, alcohols and polypeptides, are among the major constituents of root exudates, influencing the structure and function of the rhizosphere microbial community^7^. Root-associated bacteria, often referred as rhizobacteria, can form beneficial relationships with plants promoting growth and/or inducing defense^8,9^. Despite the vast amount of studies on plant growth promotion and induced systemic resistance by different rhizobacterial genera, relatively little is known to date about how rhizobacteria change plant chemistry and how these changes relate to the phenotypic changes in plant growth and resistance.

Plants produce various secondary metabolites that play key roles in the adaptation of plants to environmental changes, including tolerance to biotic stresses such as insect herbivory and pathogen infections^10–13^. Recent studies showed that a number of rhizobacteria elicit secondary metabolite accumulation, including metabolites involved in defense^8,14–17^. Such changes in plant secondary metabolism have costs associated with the biosynthesis, transport and storage of these molecules and with the competition for primary metabolites and energy needed for plant growth. The biosynthesis of primary and secondary metabolites depend on common precursors and have a trade-off at the biochemical level. Intriguingly, rhizobacteria are able to orchestrate balanced plant growth, plant defense and secondary metabolite production. Hence, investigation of how rhizobacteria influence plant primary and secondary metabolism can provide a road map of key metabolite targets that play a balancing act between plant growth and defense. Recent developments in metabolomics approaches provide valuable tools to assess the influence of rhizobacteria on the dynamics of primary and secondary metabolism and to identify key metabolite classes that have a broader impact on host plant growth and defense.

In the present study, we investigated the effects of root-colonizing strains of three *Paraburkholderia* species on the phenotypes of two Broccoli cultivars, in particular on growth and defense against the bacterial leaf pathogen *Xanthomonas campestris*. Broccoli (*Brassica oleracea* var. italica) is a crop plant known for high value natural compounds, such as glucosinolates and flavonoids^18^. In the present study, we selected two Broccoli cultivars, Coronado and Malibu, contrasting in their relative levels of glucosinolates (mainly glucoiberin, glucoraphanin and glucobrassicin). Glucosinolates are among the plant secondary metabolites involved in rhizobacteria-mediated bacterial pathogen resistance in the Brassica model species *Arabidopsis thaliana*^8^. *Paraburkholderia* is a monophyletic clade diverged from the genus *Burkholderia*^19^. A number of rhizospheric and endophytic *Paraburkhoderia* species, in particular *Paraburkholderia phytofirmans* PsJN, *P. fungorum* and *P. graminis,* have been shown to promote growth of maize, strawberry and Arabidopsis^20–22^ and suppress pathogen infections^23,24^. In addition, various *Paraburkholderia* species are typically found in the mycosphere consuming organic acids released from fungi and using the hyphae as ‘highways’ for translocation^25^. The strains of *Paraburkholderia* species used in our study are *P. graminis* PHS1 (*Pbg*), *P. hospita* mHSR1 (*Pbh*), and *P. terricola* mHS1 (*Pbt*), which exhibited plant protection against the fungal root pathogen *Rhizoctonia solani.* For *P. graminis*, we further showed that the production of sulfurous volatiles was a key mechanism in disease suppression^24^. To begin to identify the bacterial traits associated with plant growth promotion, we screened live cells, heat-killed cells, cell-free culture supernatant and volatile compounds of *Pbg* and found that for growth promotion, Broccoli requires live *Pbg* cells.

Using non-targeted metabolomics, we assessed the impact of the strains of *Paraburkholderia* species on the temporal dynamics of shoot primary and secondary metabolisms of the two Broccoli cultivars. Our results showed common and specific signatures in both primary and secondary metabolism in the two Broccoli cultivars colonized by the strains of the *Paraburkholderia* species. The results also revealed that the enhanced accumulation of soluble sugars in shoots of Broccoli upon root colonization by *Paraburkholderia* coincided with distinct changes in secondary metabolism that in turn correlate with distinctive changes in plant growth and defense. The integrated strategy adopted in this study enhanced our fundamental understanding of metabolic changes associated with rhizobacteria-mediated plant growth and defense.

## Results

### *Paraburkholderia* species promote Broccoli growth in a cultivar-dependent manner

**Figure 1:**
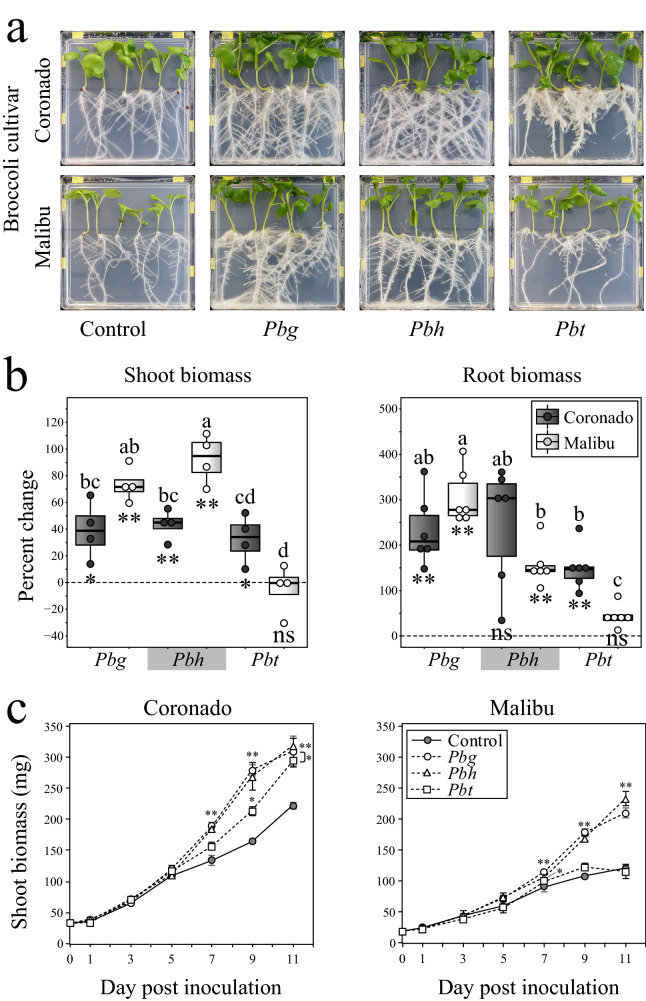
Biomass and phenotypic changes in Broccoli cultivars in response to root tip inoculation with strains of three *Paraburkholderia* species. (**a**) Pictures of MS agar plate with two Broccoli cultivars (Coronado and Malibu) at 11 days post inoculation with strains of three *Paraburkholderia* species (*Pbg*: *Paraburkholderia graminis* PHS1, *Pbh*: *P. hospita* mHSR1, and *Pbt*: *P. terricola* mHS1). (**b**) Percent changes in shoot and root biomass (mean ± standard error, n = 4 (shoot) and n = 6 (root)) of two Broccoli cultivars inoculated with the strains of the *Paraburkholderia* species. Treatments sharing the same letters are not significantly different (Two-way ANOVA, Tukey’s HSD post hoc test, *P* < 0.05). (**c**) Temporal changes in shoot biomass of two Broccoli cultivars (Coronado and Malibu) inoculated with the *Paraburkholderia* species. Asterisks in panels b and c denote significant differences from the non-treated control samples (two-tailed Student’s t test: *P < 0.05; **P < 0.01).

Root tip inoculation of the two Broccoli cultivars with strains of three different *Paraburkholderia* species led to changes in leaf color (deep green leaves), shoot biomass, root biomass and root architecture (Fig. 1a). Percent change in biomass was used as a measure to assess the growth-promoting effects of the *Paraburkholderia* species in the two Broccoli cultivars. Two-way analysis of variance (ANOVA) was conducted to assess the influence of the two independent variables (strains of *Paraburkholderia* species and Broccoli cultivars) on both shoot and root biomass. The *Paraburkholderia* species included three levels (*Pbg*, *Pbh, Pbt*) and the Broccoli cultivars consisted of two levels (Coronado, Malibu). For shoots, all interactions, except *Pbt*-Malibu, resulted in significant increases in biomass relative to the non-treated control plants, while for roots all three *Paraburkholderia* species significantly increased the biomass in both Broccoli cultivars (Fig. 1b). In general, the relative impact of *Paraburkholderia* species was up to 3 times higher for root biomass than for shoot biomass (Fig. 1b). Two-way ANOVA showed highly significant interactions between the strains of *Paraburkholderia* species and Broccoli cultivars regarding the percent changes in shoot and root biomass (Supplementary Table S1). Overall, for cultivar Coronado the percent change in shoot biomass was about 40% compared to the control, and not significantly different between the different strains of *Paraburkholderia* species, whereas in cultivar Malibu the percent change in shoot biomass was significantly higher for *Pbg* (~ 70%) and *Pbh* (~ 90%) as compared to *Pbt*. Furthermore, inoculation with *Pbh* led to a significantly higher increase in shoot biomass in cultivar Malibu than in Coronado. Regarding the percent change in root biomass, only inoculation of *Pbt* showed significant differences between the two Broccoli cultivars. As indicated above, the shoot biomass of cultivar Malibu inoculated with *Pbt* was not significantly different from the control plants (Fig. 1b). Over a period of 11 days, both *Pbg* and *Pbh-*treated Broccoli cultivars showed significantly higher shoot and root biomass from 7 days post inoculation (dpi) onwards, while *Pbt*-treated plants showed higher shoot biomass in Coronado from 9 dpi onwards (Fig. 1c).

### Relation between root colonization and plant growth promotion

**Figure 2:**
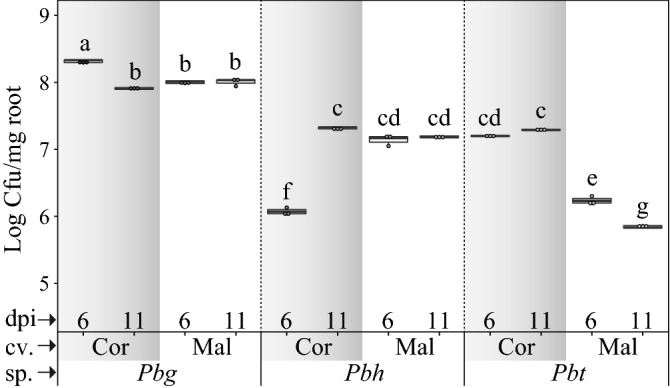
Root colonization ability of strains of three *Paraburkholderia* species (sp) for two Broccoli cultivars (cv) at 6 and 11 days post inoculation (dpi). Means of 3 replicates are shown. Treatments sharing the same letter are not significantly different based on three-ways ANOVA (dpi, Broccoli cultivars and Bacteria species, Tukey’s HSD post hoc test, *P* < 0.05). Broccoli cultivars (Cor: Coronado, Mal: Malibu) and *Paraburkholderia* species (*Pbg*: *Paraburkholderia graminis* PHS1, *Pbh*: *P. hospita* mHSR1, and *Pbt*: *P. terricola* mHS1).

The extent of root colonization of the strains of *Paraburkholderia* species was assessed for the two Broccoli cultivars at the early and late growth stages. The data was log-transformed, as they did not meet the ANOVA assumption for homogeneity of variance and normality. Three-way analysis of variance was conducted on the interaction effects of *Paraburkholderia* species strains, Broccoli cultivars and time after inoculation on root colonization. The *Paraburkholderia* species strains included three levels (*Pbg*, *Pbh* and *Pbt*), the Broccoli cultivars included two levels (Coronado, Malibu) and time after inoculation consisted of two levels (6 dpi, 11 dpi). There was a highly significant three-way interaction effect on root colonization (Supplementary Table S2). In general, *Pbg* showed significantly higher root colonization in both cultivars at both time points when compared to *Pbh* and *Pbt* (Fig. 2 and Supplementary Table S3). In addition, *Pbg* showed significantly higher root colonization in cultivar Coronado at 6 dpi (2.1 ± 0.1 × 10^8^ Cfu/mg roots) and at 11 dpi (8.1 ± 0.3 × 10^7^ Cfu/mg roots). In cultivar Malibu root colonization by *Pbg* was not significantly different between the two time points (1.0 ± 0.1 × 10^8^ Cfu/mg roots (6 dpi) and 1.0 ± 0.1 × 10^8^ Cfu/mg roots (11 dpi)). *Pbt* showed significantly lower root colonization in cultivar Malibu at both time points. Furthermore, *Pbt* on Malibu showed a significant decline in root colonization at the later time point (1.7 ± 0.2 × 10^6^ Cfu/mg roots (6 dpi) and 7.0 ± 0.4 × 10^5^ Cfu/mg roots (11 dpi)).

### *Paraburkholderia* species altered primary and secondary metabolism of Broccoli shoot

Considering the extent of strain and cultivar-dependent variations in root colonization and plant growth promotion, we investigated the systemic effect of the bacterial strains on the shoot metabolome of the two Broccoli cultivars at 6 and 11 dpi. GC–MS and LC–MS-based non-targeted metabolomics analysis of shoot extracts were performed to profile the polar primary metabolites and semi-polar secondary metabolites, respectively. The data was subjected to ANOVA with correction for multiple testing (Benjamini-Hochberg) and metabolites that were significantly different (*P* < 0.05 and fold change > 2) between at least two treatments were used for multivariate analysis. Principal Component Analysis (PCA) and Hierarchical Cluster Analysis (HCA) were used to reduce the dimensionality of the data and explore specific patterns of change in metabolome in the different plant–rhizobacteria interactions.

#### Effects of *Paraburkholderia* on shoot primary metabolism

**Figure 3:**
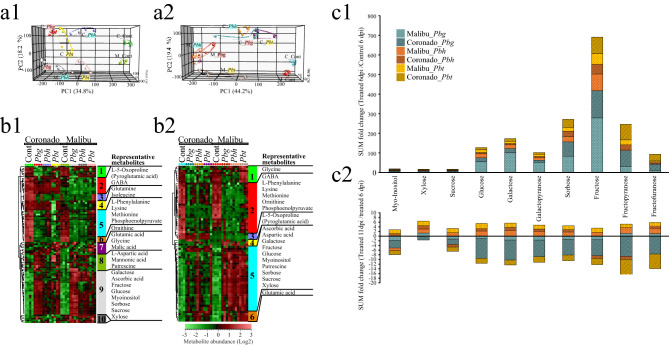
*Paraburkholderia*-mediated changes in shoot primary metabolites in two Broccoli cultivars. (**a**) Principal Component Analysis (PCA) and (**b**) Hierarchical Cluster Analysis (HCA) based on differentially regulated metabolites of the samples at 6 dpi (**1**) and 11 dpi (**2**). In the HCA, metabolite clusters are indicated by different colors. Information on the representative metabolites of each clusters is given on the right side, if the metabolites are annotated. (**c**) Impact of *Paraburkholderia* species on sugar generation (**1**) and utilization (**2**) of two Broccoli cultivars. Broccoli cultivars (Cor: Coronado, Mal: Malibu), Cont.: non-rhizobacteria treated control, *Pbg*: *Paraburkholderia graminis* PHS1, *Pbh*: *P. hospita* mHSR1, and *Pbt*: *P. terricola* mHS1.

GC–MS-based non-targeted metabolomics demonstrated that out of the 138 polar metabolites detected, 68 (50%) were significantly different between at least two treatments. At 6 dpi, PCA indicated that the first three principal components (PCs) explained 62.8% of the total variance (Fig. 3a1). The first PC (PC1), explained 34.8% of the total variance and corresponded to the effect of the three *Paraburkholderia* treatments on the metabolome of both cultivars (Fig. 3b1, Clusters 1, 5, 8 and 9). *Pbg* had the greatest impact on shoot primary metabolism of both Broccoli cultivars, while inoculation with *Pbh* and *Pbt* resulted in changes in the shoot primary metabolome in a cultivar-dependent manner. *Pbh* had greater impact on shoot primary metabolome of Malibu, while *Pbt* had greater impact on shoot primary metabolome of Coronado (Fig. 3a1). The major changes in primary metabolism induced by *Paraburkholderia* included accumulation of sugars (Cluster 9) and depletion of amino acids (Cluster 5, phenylalanine, lysine and methionine) and phosphoenolpyruvate (PEP), a key intermediate in glycolysis and gluconeogenesis. Some of the representative metabolites in cluster 8 that showed accumulation in all interactions, except in the ineffective *Pbt*-Malibu interaction, include aspartic acid, mannonic acid and putrescine. The second principal component (PC2) explained 18.2% of the total variance and resulted from metabolites that showed variation between the two cultivars. Furthermore, treatment of the two cultivars with *Pbg* and *Pbh* widened the inherent variation in the level of some of the metabolites between the two Broccoli cultivars. (Fig. 3b1, Clusters 2 and 3). Amino acids such as glutamine, oxoproline (pyroglutamic acid), GABA (γ-aminobutyric acid) and isoleucine were intrinsically higher in Coronado than in Malibu.

At the later seedling growth stage (11 dpi), the initial inoculation of the roots of the two Broccoli cultivars with the strains of the *Paraburkholderia* species continued to have substantial impact on shoot primary metabolism. PCA showed that the first three principal components explained 72.1% of the total variance (Fig. 3a2). Here, the impact of all the three strains of the *Paraburkholderia* species on the Broccoli shoot metabolome was cultivar dependent and was greater in Malibu (Fig. 3a2). The first principal component (PC1) explained 44.2% of the total variance and resulted from metabolites that were accumulated (Fig. 3b2, Cluster 5) or reduced (Cluster 2) in the *Paraburkholderia* treatments. The Broccoli metabolites that decreased upon inoculation with the strains of the *Paraburkholderia* species encompassed amino acids such as lysine, phenylalanine, methionine, the non-proteinogenic amino acids ornithine and GABA, as well as PEP. In all plant–microbe combinations, except the ineffective partnership between *Pbt*-Malibu, PEP showed 11–14 fold decreases (Supplementary excel, Table S6). Sugars and other metabolites, including ascorbic acid and aspartic acid, represented the metabolites enhanced by the *Paraburkholderia* treatments when compared to the control plants (Cluster 5). Six days after treatment with *Paraburkholderia,* sugars showed greater abundance in cultivar Malibu than in cultivar Coronado (Fig. 3b2). However, at 11 dpi, sugars in *Paraburkholderia*-treated plants showed substantial depletion in cultivar Coronado as compared to 6 dpi (Fig. 3b1), whereas in cultivar Malibu, the temporal variation in the level of these sugars was less pronounced (Fig. 3b2, Cluster 5, Supplementary Figure S3 and S4). PC2, representing 19.4% of the total variance, was associated with metabolites in cluster 1 including glycine, that were depleted in all treatment combinations except in the controls and in the ineffective partnership between *Pbt* and Malibu (Fig. 3b2, Cluster 1). Oxoproline and some other metabolites in cluster 3 were intrinsically abundant in the shoots of cultivar Coronado.

#### *Paraburkholderia* impact on Broccoli primary metabolism is highly associated with soluble sugars

As sugars are the primary drivers of plant growth, we looked into their temporal dynamics, particularly related to sugar generation and utilization in the shoots of the two Broccoli cultivars treated with the strains of the *Paraburkholderia* species. The fold change in sugar level between *Paraburkholderia* treated and control plants at 6 dpi was used as a measure of sugar generation, while the fold change in sugar level of treated plants from 6 to 11 dpi was used as a measure of sugar utilization. In control plants, the sugar levels showed no significant difference between the two Broccoli cultivars at 6 dpi (supplementary Fig. S3). In contrast, treatment with the strains of the *Paraburkholderia* species showed substantial impact on the sugar generation in shoots of both Broccoli cultivars, resulting in significant increases in the level of fructose and its derivatives, glucose, sorbose, galactose and galactopyranose at 6 dpi. Moreover, the magnitude of sugar generation showed remarkable differences between the strains of the *Paraburkholderia* species-Broccoli cultivar combinations (Fig. 3c1). *Pbg* treatment resulted in the highest sugar generation when compared to *Pbh* and *Pbt,* and this ability was significantly higher in cultivar Malibu than in Coronado. The ineffective partnership between *Pbt* and Malibu had the least impact on sugar generation. Similarly, the utilization of sugar also showed noticeable differences among the strains of the *Paraburkholderia* species-Broccoli cultivar combinations. In Coronado, *Pbg* inoculation led to greater sugar utilization when compared to cultivar Malibu. The ineffective partnership between *Pbt* and Malibu showed reduced sugar utilization when compared to the effective partnership of *Pbt* with Coronado (Fig. 3c2).

#### Effects of *Paraburkholderia* on shoot secondary metabolism

**Figure 4:**
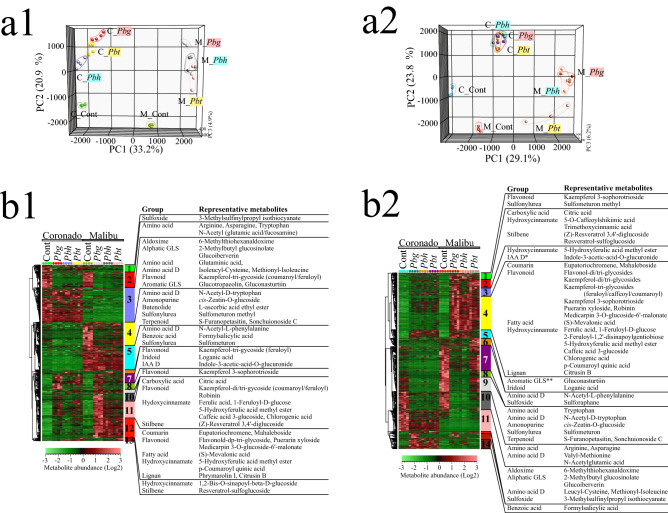
Rhizobacteria-mediated changes in the shoot secondary metabolites in Broccoli cultivars. (**a**) Principal component analysis (PCA) and (**b**) Hierarchical cluster analysis (HCA) based on differentially regulated metabolites of the samples at 6 dpi (**1**) and 11 dpi (**2**). In the HCA, metabolite clusters are indicated by different colors. Information on the representative metabolites of each clusters is given on the right side, if the metabolites are annotated. Broccoli cultivars (Cor: Coronado, Mal: Malibu), Cont.: non-rhizobacteria treated control, *Pbg*: *Paraburkholderia graminis* PHS1, *Pbh*: *P. hospita* mHSR1 and *Pbt*: *P. terricola* mHS1. *GLS = glucosinolate, **D = derivative. Some primary metabolites were also detected in the semi-polar fraction of the shoot extract, eluting at the early stage of the chromatographic separation and are listed among the secondary metabolites in panels b1 and b2.

From the 1,868 metabolites detected by LCMS, 1,386 (74%) were significantly different between at least two treatments. PCA of the metabolites at 6 dpi demonstrated distinct clustering of the samples based on the strains of the *Paraburkholderia* species-Broccoli cultivar combination (Fig. 4a1). Here PC1 explained 33.2% of the total variation and was associated with sample differences due to metabolites that were intrinsically more abundant in one of the two Broccoli cultivars (Clusters 3, 5 and 12, Fig. 4b1). Metabolites that were intrinsically more abundant in Coronado included aliphatic glucosinolates such as 2-methylbutyl glucosinolate and glucoiberverin as well as the aromatic glucosinolates glucotropaeolin and gluconasturtiin. The levels of 2-methylbutyl glucosinolate and glucoiberverin were 147 and 209 times higher in Coronado than in Malibu, respectively (Cluster 3). In Malibu, on the other hand, a number of phenolic compounds were intrinsically more abundant (Clusters 5 and 12).

The second principal component (PC2) explained 20.9% of the total variance and was associated with metabolites that were reduced (Fig. 4b1) Clusters 2 and 4) or induced (Clusters 7 and 11) by the *Paraburkholderia* treatments. Treatment of both cultivars with the *Paraburkholderia* species also widened the intrinsic cultivar variation in metabolites. Inoculation of *Pbg* had the greatest impact on the shoot secondary metabolome profile of both Broccoli cultivars, whereas the ineffective partnership between *Pbt* and Malibu had less pronounced impact on the shoot metabolome. Metabolites in cluster 2, comprising amino acids such as arginine, asparagine, tryptophan and N-acetylated glutamic acid/fucosamine, showed greater reduction in their abundance upon treatment with strains of the *Paraburkholderia* species when compared to the control. Cluster 4 encompassed metabolites that were more abundant in Malibu than in Coronado and included ascorbic acid ethyl ester, N-acetyl-tryptophan, and terpenoids putatively annotated as such as S-furanopetasitin and sonchuionoside C. The metabolites in clusters 7 and 11 were induced by all the strains of *Paraburkholderia* species and were dominated by phenolic compounds. In Malibu, inoculation of *Pbg* led to greater accumulation of flavonoids glycosides (i.e. kaempferol-di/tri-(feruloyl/coumaroyl glycosides and robinin), hydroxycinnamates (ferulic acid and its derivatives, caffeic acid derivatives such as chlorogenic acid) and indole-3-acetic-acid-O-glucuronide when compared to the other two *Paraburkholderia* species.

PC3 explained 4.9% of the total variance and was represented by *Pbg*-induced (Clusters 8 and 10) or *Pbt*-induced (Cluster 13) metabolites in both Broccoli cultivars. *Pbg*-enhanced metabolites in cluster 8 consisted of the flavonoid kaempferol 3-sophorotrioside, whereas *Pbt*-enhanced metabolites in cluster 13 included the hydroxycinnamate O-sinapoyl-beta-D-glucoside and resveratrol-sulfoglucoside, a stilbenoid.

Similarly, at 11dpi, inoculation with the strains of the *Paraburkholderia* species led to substantial changes in the shoot metabolite profiles of the two Broccoli cultivars (Fig. 4a2,b2). In the PCA, the first three PCs explained 51.1% of the total variance. The first PC, explaining 29.1% of the total variance is associated with metabolites that accumulated or were reduced in response to *Paraburkholderia* and the change in these groups of metabolites was more pronounced in Malibu cultivar (Fig. 4b2, Clusters 1, 2, 3, 4 and 5: up; 10 and 11: down). The induced metabolites in the above-mentioned clusters included flavonoids i.e. kaempferol-di/tri-glycosides (feruloyl/caffeoyl/coumaroyl), robinin, medicarpin-O-glucoside-malonate, as well as hydroxycinnamates, i.e. ferulic acid, caffeic acid and various derivatives of these metabolites. Furthermore, *Paraburkholderia* also induced coumarins such as eupatoriochromene and mahaleboside and mevalonate, a precursor of mevalonate pathways that goes into terpenoid biosynthesis. The reduced metabolites in both Broccoli cultivars included amino acids such as arginine, asparagine and N-acetylglutamic acid (Cluster 10). Meanwhile, metabolites in cluster 11 were also reduced by the *Paraburkholderia* treatment and these metabolites were intrinsically more abundant in cultivar Coronado. Some of the metabolites in cluster 11 included sulfur-containing metabolites such as 2-methylbutyl glucosinolate and glucoiberverin, derivatives of sulfurous amino acids including leucyl-cysteine and methionyl-isoleucine, as well as precursor or breakdown products of glucosinolates, for instance 6-methylthiohexanaldoxime and 3-methylsulfinylpropyl isothiocyanate.

The second PC (PC2) explained 23.8% of the total variance and was due to metabolites that were intrinsically more abundant in cultivar Malibu (Fig. 4b2, Clusters 6, 7, 8 and 9). Metabolites in clusters 6, 7, 8 and 9 showed significant reduction in all effective partnerships. Tryptophan, a building block for indolic glucosinolate and the growth hormone indole-3-acetic acid, N-acetylated amino acids including N-acetyl phenylalanine/tryptophan, terpenoids, i.e. S-furanopetasitin and sonchuionoside C, and sulforaphane, an isothiocyanate, are some of the metabolites in these clusters worth mentioning.

PC3 explained 6.2% of the total variance and was associated with yet unknown metabolites that showed *Pbg* specific alteration in both Broccoli cultivars (Clusters 13).

### *Paraburkholderia* induces systemic resistance against the bacterial leaf pathogen *Xanthomonas campestris* in a cultivar-dependent manner

As shown above, the two Broccoli cultivars exhibited inherent differences in their shoot chemistry (Fig. 4). Furthermore, treatment of the plant roots with strains of the *Paraburkholderia* species led to substantial alteration of the shoot metabolome including metabolome signatures specific to the individual combination of *Paraburkholderia* species and Broccoli cultivar (Fig. 4). Based on this, we hypothesized that the inherent and induced differences in shoot chemistry between the two cultivars could contribute to a differential defense response against leaf pathogens. To address this hypothesis, treated and control plants of the two cultivars were challenged with two bacterial leaf pathogens, i.e. *Xanthomonas campestris* pv. armoraciae P4216 (*Xca*) and *Xanthomonas campestris* pv. campestris P4014 (*Xcc*).

**Figure 5:**
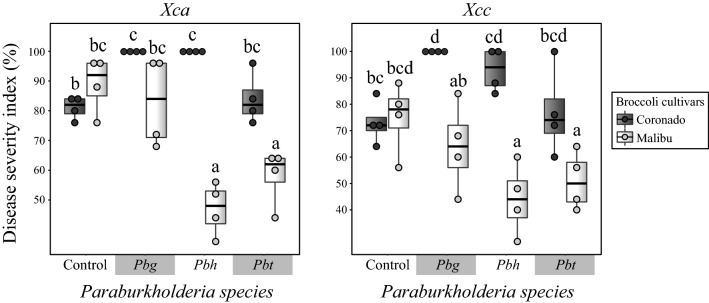
Impact of root-colonizing *Paraburkholderia* species on defense of Broccoli cultivars against two bacterial leaf pathogens. Disease severity index of two broccoli cultivars pretreated with either one of the three *Paraburkholderia* species and challenged with two bacterial leaf pathogens *Xanthomonas campestris* pv. armoraciae P4216 (*Xc*a) and *Xanthomonas campestris* pv. campestris P4014 (*Xc*c). Broccoli cultivars (Cor: Coronado, Mal: Malibu), Control: non-treated control, *Pbg*: *Paraburkholderia graminis* PHS1, *Pbh*: *P. hospita* mHSR and *Pbt*: *P. terricola* mHS1. Treatments sharing the same letter are not significantly different (Two-way ANOVA, Tukey’s HSD post hoc test, *P* < 0.05).

The interaction effect of two independent variables (*Paraburkholderia* species and Broccoli cultivars) on disease severity of the two bacterial pathogens was assessed using beta regression. The strains of the *Paraburkholderia* species included three levels (*Pbg*, *Pbh* and *Pbt*) and the Broccoli cultivars consisted of two levels (Coronado and Malibu). There was a highly significant interaction effect of the strains of the *Paraburkholderia* species and Broccoli cultivars on disease severity on both *Xanthomonas* pathovars (Supplementary Table S4). No significant inherent variation in disease severity was observed between the two Broccoli cultivars when control plants were challenged with the two bacterial pathogens (Fig. 5). However, treatment of the roots with *Paraburkholderia* led to a clear reduction or enhancement of disease severity. For example, treatment with *Pbg* and *Pbh* enhanced disease severity by 18–28% in cultivar Coronado challenged by both bacterial pathogens, whereas *Pbh* and *Pbt* significantly reduced the disease severity in cultivar Malibu challenged by *Xca* (47% and 30%, respectively) and *Xcc* (33% and 28%, respectively) (Fig. 5).

## Discussion

Our results show that strains of root-colonizing *Paraburkholderia* species alter shoot primary and secondary metabolism of Broccoli seedlings, promote their growth and induce systemic defense against the bacterial leaf pathogen *Xanthomonas campestris*. The magnitude of the alteration of these traits is dependent on the *Paraburkholderia* strain-Broccoli cultivar combination. The widely accepted “growth-defense tradeoff” concept asserts that activation of plant defense comes at the expense of plant growth due to resource limitations^26^. Here we showed that some rhizobacterial *Paraburkholderia* strains can promote plant growth and at the same time induce plant defense against biotic stress factors. Rhizobacteria-induced defense is considered to have lower cost when compared to the activation of direct defense^27,28^. However, assessing some key physiological processes such as seed production, number of flowers, pollen quality and number, and plant growth does not necessarily explain the energy and carbon costs associated with defense priming. Hence, to begin to understand the underlying mechanisms by which rhizobacteria can promote growth and simultaneously prime the plant’s defense without compromising plant fitness, a comprehensive investigation of the temporal changes induced in the host metabolome network is needed.

**Figure 6:**
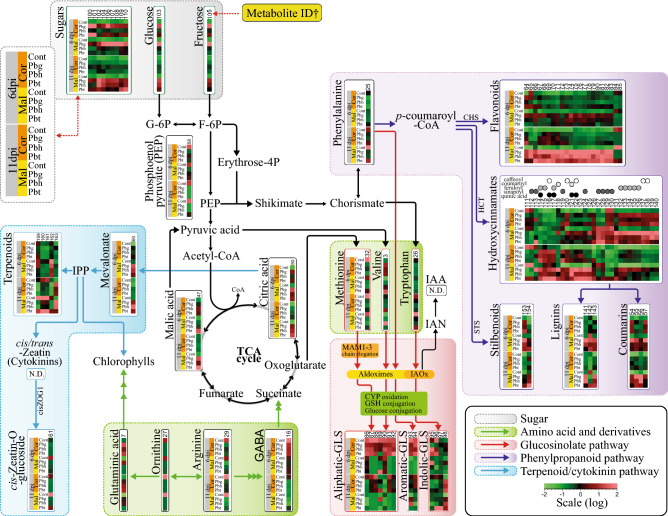
Alteration of core primary and secondary metabolite pathways by strains of *Paraburkholderia* species in Broccoli. The metabolite pathways are organized as modules inside different colored boxes and the abundance of the significantly altered metabolites is depicted by the heat map, with each cells representing the abundance of a metabolite of a sample for *Paraburkholderia* species-Broccoli cultivar and time combinations. The metabolite ID corresponding to each metabolites is indicated at the top of the heat map and detailed information on the identity of the individual metabolites is provided in the supplementary excel Table S9. G-6P (Glucose 6-phosphate), F-6P (Fructose 6-phosphate), CHS (Chalcone synthase), cisZOG1 (Cis-zeatin O-glucosyltransferase 1), CYP (Cytochrome P450), GABA (γ-aminobutyric acid), GLS (Glucosinolates), GSH (Glutathione), HCT (hydroxycinnamoyl-CoA shikimate/quinate hydroxycinnamoyl transferase), IAA (indole-3-acetic acid), IAN (indole‐3‐acetonitrile), IAOx (indole-3-acetaldoxime), IPP (isopentenyl pyrophosphate), MAM (methylthioalkylmalate synthase), and STS (stilbene synthase). Broccoli cultivars (Cor: Coronado, Mal: Malibu), Cont.: non-rhizobacteria treated control, *Pbg*: *Paraburkholderia graminis* PHS1, *Pbh*: *P. hospita* mHSR1, and *Pbt*: *P. terricola* mHS1. Multiple-headed arrows indicate hidden intermediate processes in the pathways.

The two Broccoli cultivars used in this study showed inherent differences in their shoot metabolome profile: phenolics were more abundant in cultivar Malibu and glucosinolates and other sulfur-containing compounds showed higher abundance in cultivar Coronado. The *Paraburkholderia* strains exerted a substantial impact on primary and secondary metabolism at both early and later stages of Broccoli seedling growth. Their biggest impact on primary metabolism was reflected in the generation and utilization of soluble sugars, and both parameters showed significant variation across the strains of the *Paraburkholderia* species-Broccoli cultivar combinations (Fig. 3c). All combinations, except *Pbt*-Malibu, resulted in an effective partnership, i.e. plant growth promotion. These effective partnerships showed high soluble sugar generation at the early stage of seedling growth and high sugar utilization at the later stage of seedling growth, whereas the ineffective partnership between Malibu and *Pbt* showed a lower sugar generation and utilization. Soluble sugars are fuel for plant growth and for the biosynthesis of secondary metabolites involved in defense^29–31^. Moreover, soluble sugars such as galactose, glucose, sorbose, fructose, sucrose and xylose have been reported to be effective chemotaxis agents for bacteria^32–34^. In general, low sugar concentration promotes ‘source’ activities such as photosynthesis, nutrient mobilization and export, while high sugar level enhances ‘sink’ activities such as growth and sugar storage^35^. Hence, the enhancement of the levels of plant soluble sugars such as sorbose and fructose by beneficial *Paraburkholderia* strains can be considered as one of the metabolic signatures of an effective partnership. Furthermore, under effective partnership, there was significant depletion (> 11-fold) of PEP, a key substrate for the TCA cycle and the shikimate pathway (Fig. 6), whereas PEP depletion was only about twofold in the ineffective partnership. Furthermore, greater depletion of GABA under the effective partnership suggests catabolism of GABA to succinyl semialdehyde followed by its conversion to succinate to feed the greater demand of pyruvate in the TCA cycle. Key intermediates in the TCA cycle such as citric acid and malic acid showed increased abundance under effective partnerships. In the TCA cycle, citrate is converted to malate and used in the mitochondria for energy production^36^. Hence, these observations suggest that the beneficial *Paraburkholderia*-Broccoli interactions most likely require a greater demand for carbon and energy needed for enhanced growth and defense priming.

The relationship between root colonization density of the *Paraburkholderia* strains and the corresponding growth promotion effect on Broccoli cultivars was not linear. Past studies on dose–response relationships in bacteria-plant interactions indicated that increasing the rhizobacteria densities beyond the threshold density required to induce a given phenotype did not further enhanced the impact on the phenotype ^37,38^. The *Pbg*-Malibu interaction showed the highest root colonization and had the highest impact on shoot fructose level (~ > 280 folds). In contrast, *Pbt*-Malibu and *Pbh*-Coronado interactions showed the lowest root colonization levels and had the lowest impact on shoot fructose generation at the early stages of seedling growth. Interestingly, *Pbg* also showed the biggest impact on secondary metabolism in general, and on phenolic compounds accumulation in particular, when compared to *Pbh* and *Pbt* at 6 dpi (Figs. 4a1,b1, 6). This suggests that the effects of elevated levels of soluble sugars might not only be limited to plant growth but also extended to secondary metabolites biosynthesis. For example, fructose is the primary substrate for fructose-6-phospate, a key substrate for the biosynthesis of both PEP and erythros-4-phosphate (Fig. 6). The latter two intermediates are channeled to the shikimate pathway that bridges carbohydrate metabolism to biosynthesis of aromatic primary and secondary metabolites^29^. The shikimate pathway provides all the important precursors for the biosynthesis of phenolic compounds including hydroxycinnamates, flavonoids, stilbenoids, coumarins and lignins, that all showed significant accumulation in plants treated with *Paraburkholderia* (Figs. 4b, 6). A study on the *pho3* mutant of Arabidopsis, which accumulates soluble sugars to high levels, showed large increases in the expression of transcription factors and enzymes involved in anthocyanin biosynthesis^39^. Another study, on quinoa cotyledons, also showed accumulation of both fructose (~ tenfold) and flavonoids in response to UV-B radiation^40^. Considering our results and these previous findings by others, we postulate that one of the key mechanisms by which rhizobacteria modulate host secondary metabolism is by soluble sugar generation.

In addition to enhancement of key precursors for growth and secondary metabolite biosynthesis, treatment of Broccoli roots with *Paraburkholderia* also induced metabolite remobilization. The metabolite re-direction involved both suppression of resource competing metabolite pathways, such as amino acids, and rechanneling of existing primary metabolite-derivatives and other secondary metabolites to other metabolite pathways. For example, aromatic glucosinolates, amino acids and their derivatives, and some terpenoids showed more depletion under effective partnerships (Figs. 4b2, 6). Hence, re-direction of existing metabolites towards specific metabolite pathways could be an additional strategy used by rhizobacteria to reduce the costs of de novo biosynthesis of metabolites.

Our results also showed that rhizobacteria-mediated reorganization of the host metabolome landscape affected not only plant growth but also the defense response of Broccoli cultivars to the bacterial leaf pathogen *Xanthomonas*. In general, inoculation of the strains of the *Paraburkholderia* species showed greater suppression of pathogen proliferation in cultivar Malibu that intrinsically accumulates higher levels of phenolic compounds (Fig. 5). The phenolic pathway in leaves appears to be the central target by the *Paraburkholderia* species and was altered more in Malibu than in Coronado (Fig. 6). All metabolite classes belonging to this pathway including flavonoids, hydroxycinnamates, stilbenoids, coumarins and lignins showed substantial accumulation upon treatments with the strains of the three *Paraburkholderia* species (Fig. 6). These metabolites have direct antimicrobial effects and/or act as a physical barrier against pathogenic microorganisms^41,42^. For example, hydroxycinnamic acids and flavonoids were shown to negatively affect the disease symptom development in Chinese cabbage challenged by *Xanthomonas campestris* pv. campestris (*Xcc*)^43^. Of all the *Paraburkholderia* species-Broccoli cultivars combinations, treatment of roots of cultivar Coronado with *Pbg* and *Pbh* resulted in higher disease severity (Fig. 5). However, we could not trace metabolites that are specifically induced or reduced in Coronado cultivar that is primed with *Pbg* and *Pbh*. Priming is a complex process that alters various components of the plant immune system, including key plant hormones with a role in defence signalling (such as SA, JA and Ethylene), pathogenesis-related proteins and phytoalexins. Priming might have resulted in broad-spectrum/cross resistance or compromised resistance that is effective only against certain group of microbes/herbivores^44,45^. Hence, the case of Coronado cultivar primed with *Pbh* and *Pbg* might fall under compromised resistance, and perhaps those plants might be resistant against other fungal/bacterial pathogens. Moreover, induced resistance against a pathogen might not exclusively be explained by the associated change in the metabolome as priming alters wide ranges of components of the plant immune machinery. Even the combined untargeted metabolite profiling used in our study does not cover all the metabolites that are produced during the interaction of the plants with the rhizobacteria. For example, metabolites of paramount importance in plant–microbe interactions, such as phospholipids, other non-polar metabolites and volatile organic compounds were not analysed in our study. Profiling of such a wider array of metabolite groups could provide other metabolite signatures of induced disease susceptibility.

Defense priming is not a low-cost defensive measure but could cost substantial amounts of energy and carbon resources. This is shown by the massive accumulation of phenolics and other defensive compounds in plants treated with the strains of the *Paraburkholderia* species even before the plants were challenged with the bacterial leaf pathogens. The integrated primary and secondary metabolome profiling of primed plants suggests that rhizobacteria could avert the negative impact of defense priming on the host fitness by generating substantial amounts of soluble sugars and remobilizing other metabolites to accommodate for the high energy and carbon skeleton demand associated with growth and defense priming. This suggests that defense costs can be regulated, if resources are not limiting. This hypothesis aligns with studies that showed that the inevitability of growth-defense trade-off occurs primarily under resource-limiting conditions^46,47^.

It should be noted that the physiological state of the plants grown on MS media in our experimental design may be different from that of Broccoli plants grown in agricultural fields. Nevertheless, we used proper controls for all experiments and the effect of the rhizobacteria on different phenotypic and metabolomic traits were evaluated by comparing them with mock-inoculated plants. Our data indicated that treatment of the plants with the strains of the *Paraburkholderia* species alter the baseline growth level of the plants, their metabolism and their response to biotic stress in a *Paraburkholderia* strain-Broccoli cultivar specific manner. Hence, our comprehensive metabolomic and phenotypic analyses provided the first essential step in understanding the temporal changes in plant metabolism induced by root-colonizing bacteria and their association with plant growth promotion and plant defense. The observed and discussed metabolic hallmarks associated with effective partnerships between the *Paraburkholderia* strains and Broccoli cultivars potentially play a regulatory role in carbon and energy economy of the plant to ensure sustained growth and defense. Further extensive transcriptional and mutational analyses of the bacterial strains and its host plant as well as greenhouse and field trials will be the next steps for understanding of the functional importance of the specific changes in phenotype and metabolome induced by these rhizobacteria. These elaborate analyses also involving the model Brassica plant species Arabidopsis could lay a blueprint for a new microbiome-based plant breeding strategy to produce crops in which high yielding and stress-resilience go hand in hand.

## Materials and methods

### Bacterial strains and culture conditions

The epiphytic rhizobacterial strains *Paraburkholderia graminis* PHS1 (*Pbg*), *P. hospita* mHSR1(*Pbh*), and *P. terricola* mHS1 (*Pbt*) used in this study were originally isolated from rhizospheric soil of *Beta vulgaris* grown in *Rhizoctonia solani* suppressive soil^24^. Cultures of *Paraburkholderia* species were maintained in Luria Bertani (LB)-medium (Lennox, Carl Roth) at 25 ℃. After incubation for 16 h, bacteria cells pellets were recovered by centrifugation. These pellets were then washed three times with 10 mM MgSO_4_ and resuspended in 10 mM MgSO_4_ to a final density of OD_600_ = 1.0 (~ 10^9^ cells per ml).

### Plant materials and growth conditions

Seeds of two Broccoli cultivars (*Brassica oleracea* var. italica), Coronado and Malibu, were kindly provided by Bejo Seeds (Warmenhuizen, The Netherlands). The seeds were surface sterilized for 30 min by immersing them in 1% (v/v) sodium hypochlorite amended with 0.1% (v/v) of Tween 20, and rinsed three times with ample sterile distilled water. Thereafter, six seeds were sown on 100 X 100 mm square petri dishes containing 50 ml of half-strength Murashige and Skoog (0.5 X MS) agar media with 0.5% sucrose (w/v) and 1.2% plant agar (w/v). Five days after germination and vegetative growth in petri dishes, the root tips of the seedlings from the two Broccoli cultivars were inoculated with 2 µl cell suspension (± 10^9^ cells per ml) of each strain of the three *Paraburkholderia* species. Plants treated with 2 µl 10 mM MgSO_4_ served as controls. The determination of the initial density was based on our earlier studies on plant growth promotion and induced systemic resistance by *Pseudomonas fluorescens* SS101^8,9^. Moreover, comparison of different initial inoculum density of *Pbg* on Arabidopsis roots showed no significant influence on the final root bacterial density and on plant growth promotion (data not shown). The plates with the control and inoculated plants were then sealed and incubated in a climate chamber (21 °C / 21 °C day/night temperature; 180 µmol light m^-2^ s^-1^ at plant level during 16 h/d; 70% relative humidity) until harvest (11 days post inoculation). Temporal changes in shoot fresh biomass were measured every two days until harvest.

### Rhizobacteria root colonization assay

Bacterial root colonization was determined at 6 and 11 dpi for each of the three *Paraburkholderia* species on each of the two Broccoli cultivars. Briefly, treated roots were collected at 6 and 11 dpi and placed in sterile 50 mL Falcon tubes and its biomass was measured. Then the root samples were vortexed (60 s) in 10 mM MgSO_4_, sonicated (60 s), and again vortexed (15 s) to re-suspend the bacteria adhering to the root. The suspensions were serial dilution plated onto PSA plates containing 100 µg ml^-1^ delvocid (DSM) to inhibit fungal growth. Plates were incubated at 25 °C in the dark for 3 days, colonies were counted and the number of colony-forming units (Cfu) per gram of root fresh weight was calculated.

### Plant phenotyping

Fresh biomass of the Broccoli shoots was measured to determine the effect of the rhizobacteria on plant growth. For Broccoli, shoot fresh biomass from the respective treatments was weighed every two days after bacterial inoculation until the last harvest at 11 dpi. The average weight of 6 Broccoli seedlings was considered as one independent biological replica. The roots were carefully removed from the MS-agar and washed with distilled water to eliminate adhering agar, blotted dry on filter paper and their fresh weight was recorded.

### Induced resistance assays

To assess the impact of *Paraburkholderia* species on induced resistance, the two Broccoli cultivars were inoculated with the three *Paraburkholderia* species and grown for 11 days. Thereafter, leaves were inoculated with the bacterial leaf pathogens *Xanthomonas campestris* pv. Armoraciae P4216 (*Xc*a) and *Xanthomonas campestris* pv. Campestris P4014 (*Xc*c). To do that, *Xc*a and *Xc*c were cultured in LB-medium at 25 ℃. After 16 h, bacterial cells were washed following a similar procedure for *Paraburkholderia* as described above. A 2 µl suspension of *Xc*a or *Xc*c (~ 1 X 10^9^ cell per ml) was inoculated on the first true leaf of the Broccoli seedlings after scratching the leaf surface with sterile 20 µl pipet tips. Ten days after pathogens challenge, disease severity on shoot was assessed by determining the migration of lesion from the inoculation spot to the other parts of the shoot based on a 0-to-5 ordinal scale as shown in supplementary Fig. S2: 1 = no necrosis or migration, 2 = necrosis of the treated leaf, 3 = migration of the lesions to the leafstalk of the treated leaf, 4 = necrotic or water-soaked lesions of the neighboring (nontreated) leaf, and 5 = infection of the entire seedling. Severity values were converted to 0 to 100 Disease severity index (DSI) according to the following equation used by^48^. DSI (%) = ∑ (scores of all plants)/ [Maximum disease score x (total number of plants)] × 100.

### Plant metabolomics

#### Sample collection

Shoots from the control plants and plants treated with three *Paraburkholderia* species were harvested at 6 and 11 dpi. For each plant cultivar x *Paraburkholderia* combination, 4 biological replicates of 6 plants each were considered. Briefly, shoots were snap frozen in liquid nitrogen and ground to fine powder under continuous cooling through addition of liquid nitrogen and the samples were kept at -80 ℃ until further use.

#### Polar primary metabolite extraction and analysis

Polar primary metabolite sample preparation was performed as described by Carreno-Quintero et al.^49^. A total of 1.4 mL of methanol containing ribitol (0.2 mg/mL) as an internal standard was added to a 2 mL Eppendorf tube containing a total of 200 mg Broccoli leaf powder. After vortexing (10 s) and shaking in a thermomixer at 950 rpm for 10 min, the samples were centrifuged at maximum speed for 10 min. 500 µL of the supernatant was transferred to a new 2 mL Eppendorf tube and 370 µl of chloroform and 750 µl of distilled water were added. The mixture was vigorously mixed by vortexing and centrifugation for 10 min at maximum speed (14,000 rpm) and 50 µl of the upper polar phase was transferred to an insert in a 2 mL vial. The solvent was then vacuum dried (speedvac) for 16 h at room temperature and sealed under an argon atmosphere. The dried samples were derivatized online as described by Lisec et al.^50^ using a Combi PAL autosampler (CTC Analytics). Initially, 12.5 µl methoxyamine (20 mg mL^-1^ pyridine) was added to each of the samples and incubated for 30 min at 40 °C under agitation. The samples were then derivatized for one hour by adding 17.5 µl of *N*-methyl-*N*-(trimethylsilyl) trifluoroacetamide (MSTFA). An alkane mixture (C11-C21 and C24-C33) was added to determine the retention indices of metabolites. The derivatized samples were analyzed by a GC-TOF–MS system consisting of an Optic 3 high-performance injector (ATAS) and an Agilent 6890 gas chromatograph (Agilent Technologies) coupled to a Pegasus III time-of-flight mass spectrometer (Leco Instruments). A 2 µL of each sample was subjected to the injector at 70 °C using a split flow of 19 mL min^-1^. The chromatographic separation was performed using a VF-5 ms capillary column (Varian; 30 m × 0.25 mm × 0.25 mm) including a 10-m guardian column with helium as carrier gas at a flow rate of 1 mL min^-1^. The temperature was isothermal for 2 min at 70 ℃, followed by a 10 ℃ min^-1^ ramp to 310 ℃, and was held for 5 min. The transfer line temperature was set at 270 ℃. The column effluent was ionized by electron impact at 70 eV. Mass spectra were acquired at 20 scans s^-1^ within a mass-to-charge ratio range of 50 to 600 at a source temperature of 200 ℃. A solvent delay of 295 s was set. The detector voltage was set to 1,400 V.

#### Semi-polar secondary metabolite extraction and analysis

For extraction of semi-polar secondary metabolites, 300 µL of 99.89% methanol containing 0.13% (v/v) formic acid was added to 100 mg plant material in 2 ml round bottom Eppendorf tubes, and sonicated for 15 min followed by centrifugation for 15 min at 20,000 g. The supernatants containing predominantly the semi-polar metabolites were transferred to 96-well filter plate (AcroPrepTM, 350 µL, 0.45 µm, PALL), and vacuum filtrated into the 96-deep-well autosampler plates (Waters) using a Genesis Workstation (Tecan Systems).

LCMS profiling of these semi-polar extract was performed using an Ultimate 3000 U-HPLC (Dionex) coupled to a Q-Exactive Plus Orbitrap FTMS (Thermo), as described recently^51^. A Luna C18 column (2.0 X 150 mm, 3 µm; Phenomenex) maintained at 40 °C and a 45 min linear gradient of 5–35% acetonitrile in 0.1% formic acid in water at a flow rate of 0.19 ml min^-1^ were used to separate the compounds present in 5 µl of each extract^52^. Full scan MS data were generated with electrospray in switching positive/negative ionization mode at a mass resolution of 35,000 (FWHM at m/z 200) in the range of *m/z* 95–1350. Subsequent MS/MS experiments for identification of selected metabolites were performed with separate positive or negative electrospray ionization at a normalized collision energy of 27 and a mass resolution of 17,500. Some primary metabolites were also detected in the semi polar fraction of the shoot extract eluting at the early stage of the chromatographic separation and are listed among the secondary metabolites in the Fig. 4b1,b2, supplementary Table S7 and S8.

### Data processing and analysis

#### Preprocessing

The raw data from GC-TOF–MS analysis was preprocessed by Chroma TOF software 2.0 (Leco Instruments). Baseline-corrected mass features of the raw GC-TOF–MS and LCMS data were extracted and aligned using Metalign software^53^. The mass features were considered as a reproducible signal if they were detected in at least 3 biological replicates of any treatment with a signal intensity at least 3 times higher than the background noise value calculated by Metalign. Then, mass features originating from the same metabolite, as generated in the ion source, were clustered based on similarities in both retention time and relative abundance across all measured samples, using MSClust software^54^. This software removes metabolite signal redundancy and generates so-called centrotypes, representing reconstructed putative metabolites including their in-source mass spectra. The relative abundance of each compound in a given sample is represented by the Measured Ion Counts (MIC), which is the sum of the ion count values (corrected by their centrotype membership) for all clustered ions. The samples were batch corrected to reduce batch effect of large series of samples during the run according to^55^. The relative intensity of the detected metabolites by GC-TOF–MS was normalized to the internal standard, ribitol.

### Multivariate analysis

The preprocessed data from both analysis platforms were log-transformed and scaled and used for ANOVA. Metabolites that were significantly different between at least two treatments with a fold changes > 2.0 were used to perform Principal component analysis (PCA) and hierarchical cluster analysis (HCA). The HCA was performed using Pearson’s correlation coefficient and Unweighted Pair Group Method with Arithmetic Mean (UPGMA).

### Metabolite identification

#### GC-TOF–MS

To automatically identify metabolites, the reconstructed mass spectra file resulting from the MSClust software was introduced to NIST MS search software (v 2.2) with both the Wiley spectral library and an in-house library constructed using standards, followed by comparison of the reported and observed retention indices as determined by a series of alkanes. Metabolite annotations of selected compounds were manually curated.

#### LC–MS

Annotation of differentially regulated metabolites was performed based on selection of pseudomolecule ions from the masses in the MSClust-reconstructed metabolites, first by matching their accurate masses plus retention times to previously reported metabolites present in Arabidopsis and Broccoli on the same LC–MS system and similar chromatographic conditions^8,56^. If compounds were not yet present in this experimentally obtained database, detected masses were matched with compound libraries, including Metabolomics Japan (www.metabolomics.jp), the Dictionary of Natural Products (http://dnp.chemnetbase.com), KNApSAcK (http://kanaya.naist.jp/KNApSAcK), and Metlin (http:// metlin.scripps.edu/) using a maximum deviation of observed mass from calculated mass of 5 ppm. The identity of potential candidate metabolites was further verified from the MSMS data using the online Magma tool^57^ that compares the Insilico fragmentation patterns of a given metabolite to the experimentally obtained MSMS fragmentation pattern.

### Statistical analysis

The relative changes in shoot biomass, root biomass in the combinations of the two Broccoli cultivars and *Paraburkholderia* species was analyzed with R Studio software (Version 3.6.1). First, the normality and homogeneity of variance of the data was assessed and when the two assumptions were not met, the data was transformed using Box-Cox or log transformation using a package MASS. Differences were tested by two-way analysis of variance (ANOVA). A Tukey-HSD test was used to separate group mean values when the ANOVA was significant at *p* < 0.05. The ANOVA table is shown in Supplementary Material, Table S1 Differences in phenotypic parameters between the rhizobacterial treatments and non-treated controls were assessed by Student’s *t*-Test.

## Supplementary Information

Supplementary Information 1.

Supplementary Information 2.

Supplementary Information 3.
