# Supplementary material for: Impact of root-associated strains of three Paraburkholderia species on primary and secondary metabolism of Brassica oleracea
Source: Sci Rep. 2021 Feb 2;11:2781. doi: 10.1038/s41598-021-82238-9 (PMC7854645; doi:10.1038/s41598-021-82238-9)
Supplement: Supplementary file 2 — Supplementary Information 2. [file 41598_2021_82238_MOESM2_ESM.docx]

Impact of root-associated *Paraburkholderia* species on primary and secondary metabolism of *Brassica oleracea*

Je-Seung Jeon^1,2^, Natalia Carreno-Quintero^1,3^, Henriëtte D.L.M. van Eekelen^4^, Ric C.H. De Vos^4^, Jos M. Raaijmakers^1,2^ and Desalegn W. Etalo^1*^

^1^Netherlands Institute of Ecology NIOO-KNAW, Department of Microbial Ecology, Wageningen, 6708 PB, Netherlands. ^2^Institute of Biology, Leiden University, Leiden 2333 BE, The Netherlands; ^3^KeyGene, Wageningen, 6708PW, The Netherlands; ^4^Wageningen Plant Research, Bioscience, Wageningen, 6708 PB, The Netherlands


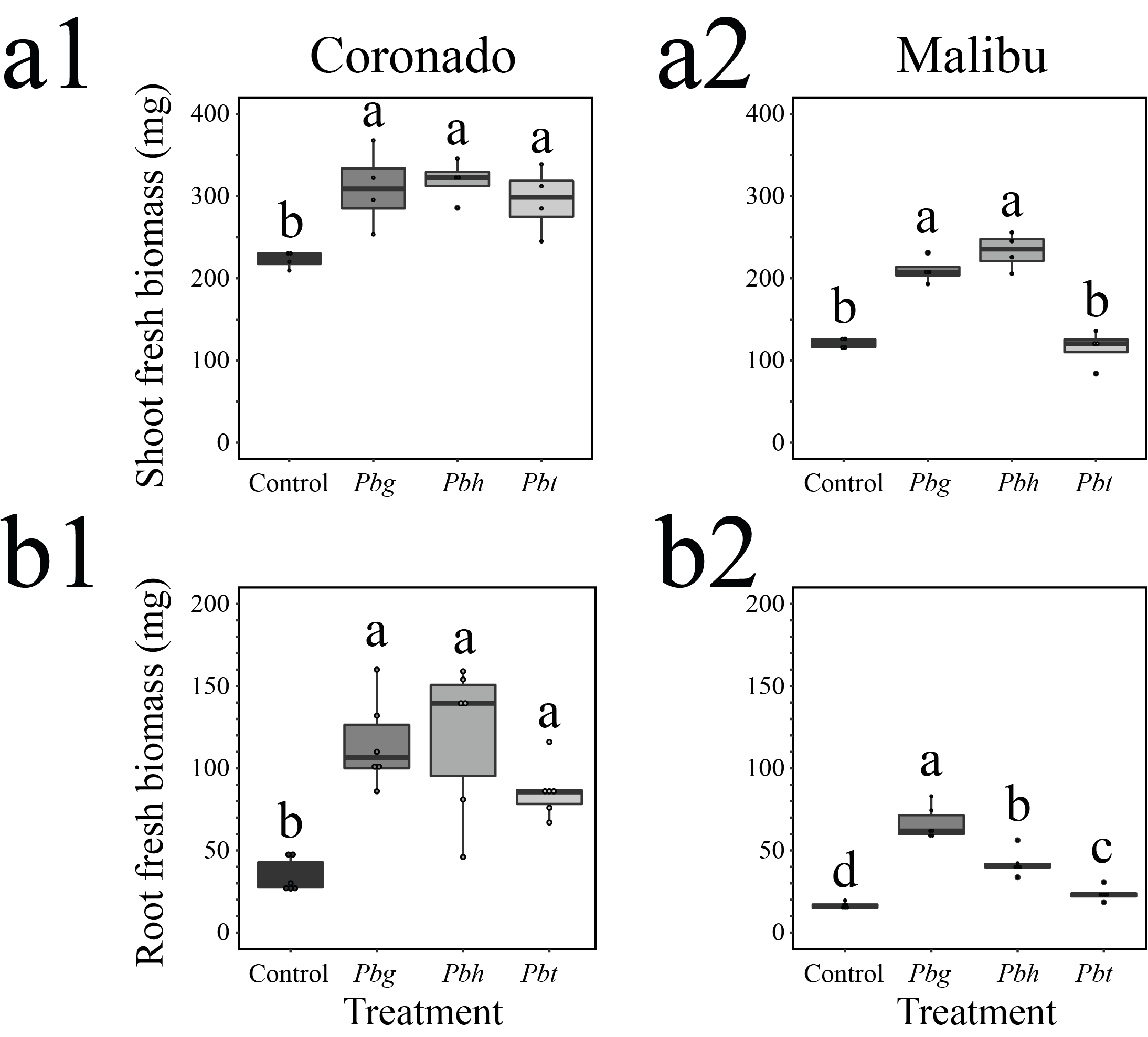


Supplementary Figure S1. Absolute biomass changes of shoot (**a**) and root (**b**) of Coronado (**1)** and Malibu (**2)** at 11 dpi. *Pbg*: *Paraburkholderia graminis*, *Pbh*: *P. hospita*, and *Pbt*: *P. terricola.* Different letters show statistically different among the treatments (One-way ANOVA, Tukey’s HSD *post hoc* test, *P* < 0.05).


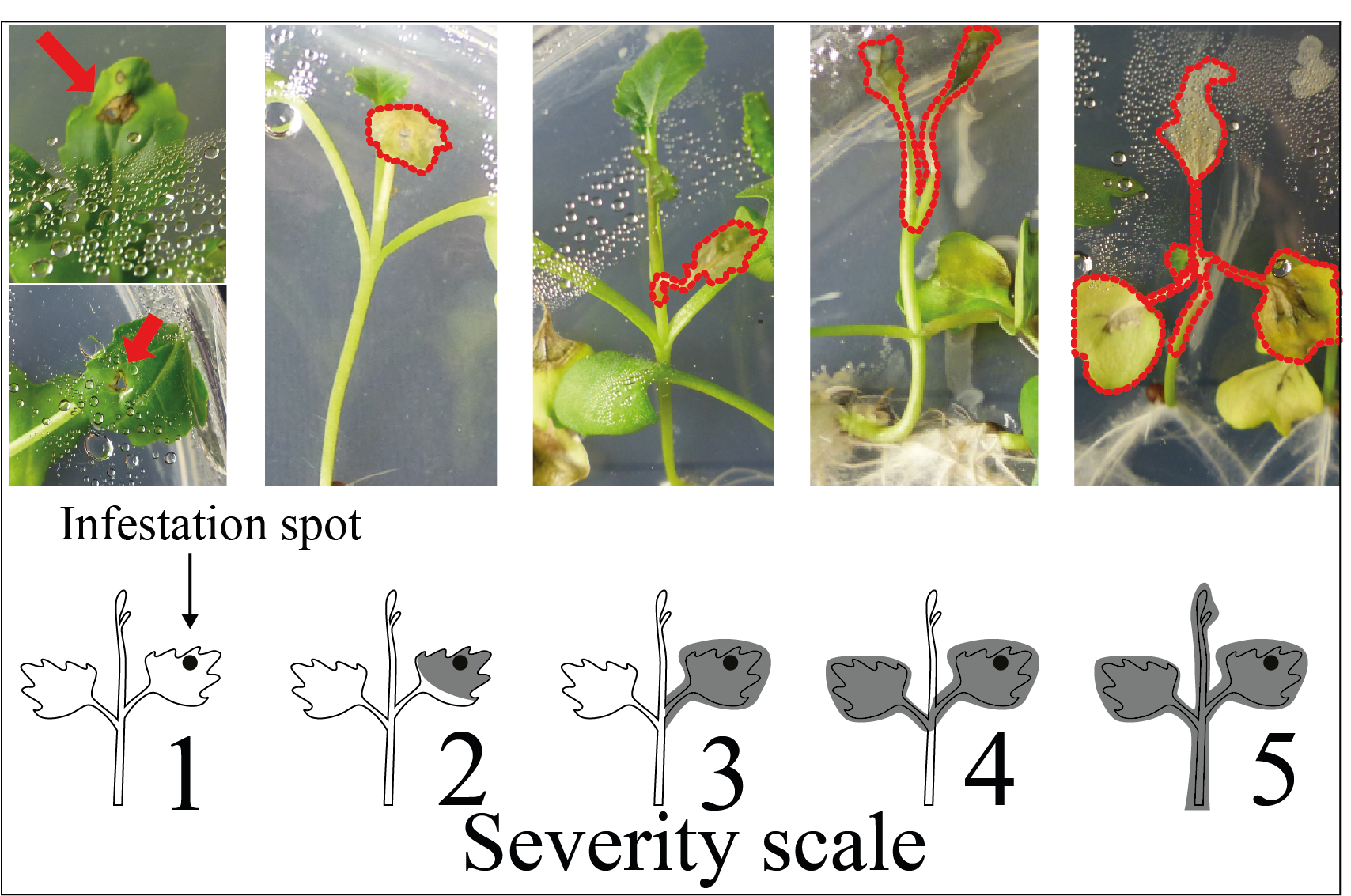


Supplementary Figure S2. Disease severity scale. For the severity (**c**), each Broccoli seedlings from four biological replicates were individually scored (n = 20). Disease severity was scored on a scale from 0-5, where 1 = no necrosis or migration, 2 = full infection of the treated leaf, 3 = migration to the leafstalk of the treated leaf, 4 = infection of the neighboring leaf, and 5 = infection of the entire seedling.


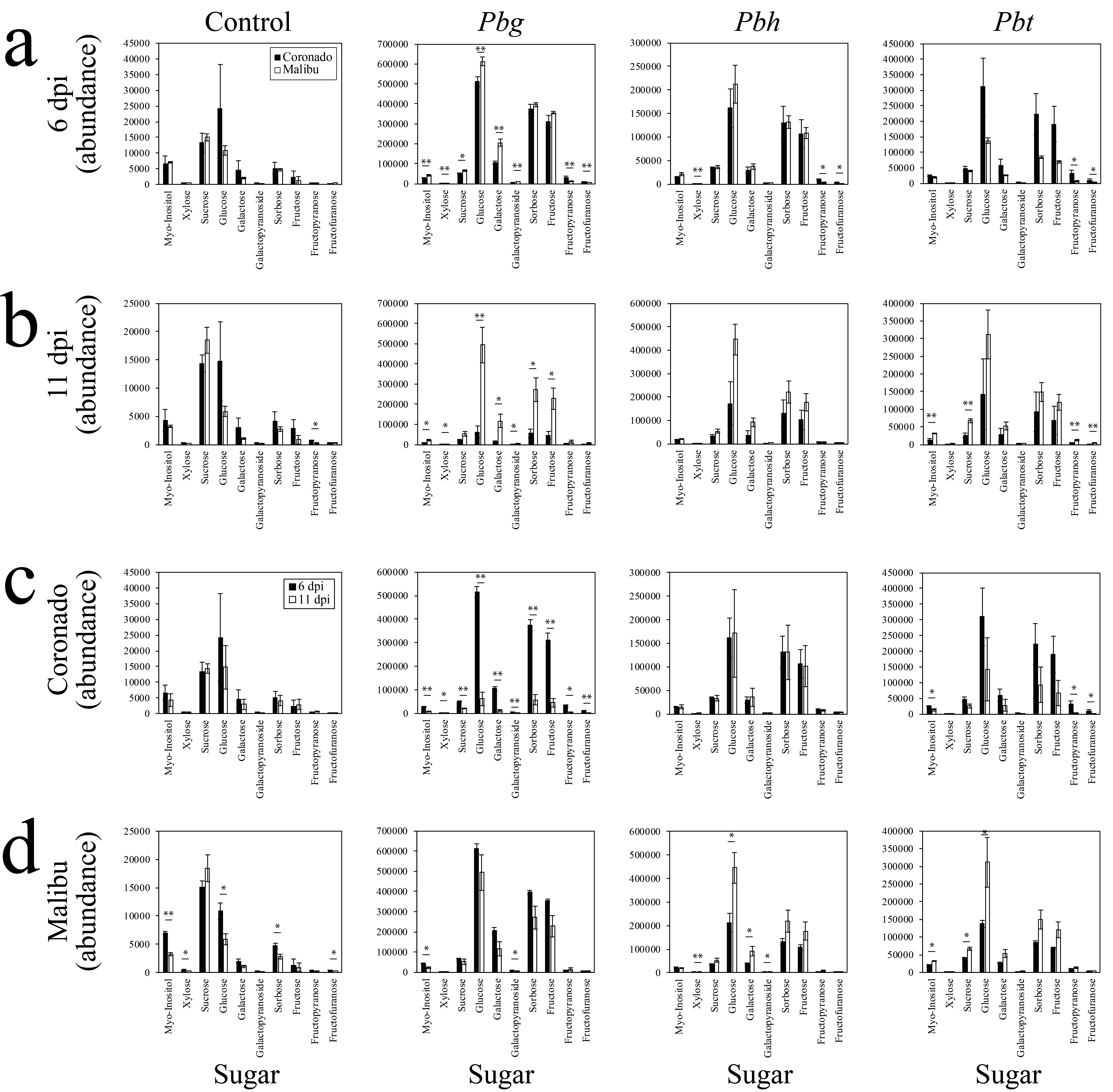


Supplementary Figure S3. Absolute abundance of soluble sugar in two Broccoli cultivars upon *Paraburkholderia* inoculation at two time points (6 and 11 dpi). Comparison of sugar abundance between two cultivars at 6 dpi (**a)** and at 11 dpi (**b**). Comparison of sugar abundance between two time points in Coronado (**c**) and Malibu (**d**). *Pbg*: *Paraburkholderia graminis*, *Pbh*: *P. hospita*, and *Pbt*: *P. terricola*. Asterisks denote statistical differences between Broccoli cultivars, Coronado and Malibu (two tailed Student’s t test): * P < 0.05; ** P < 0.01.


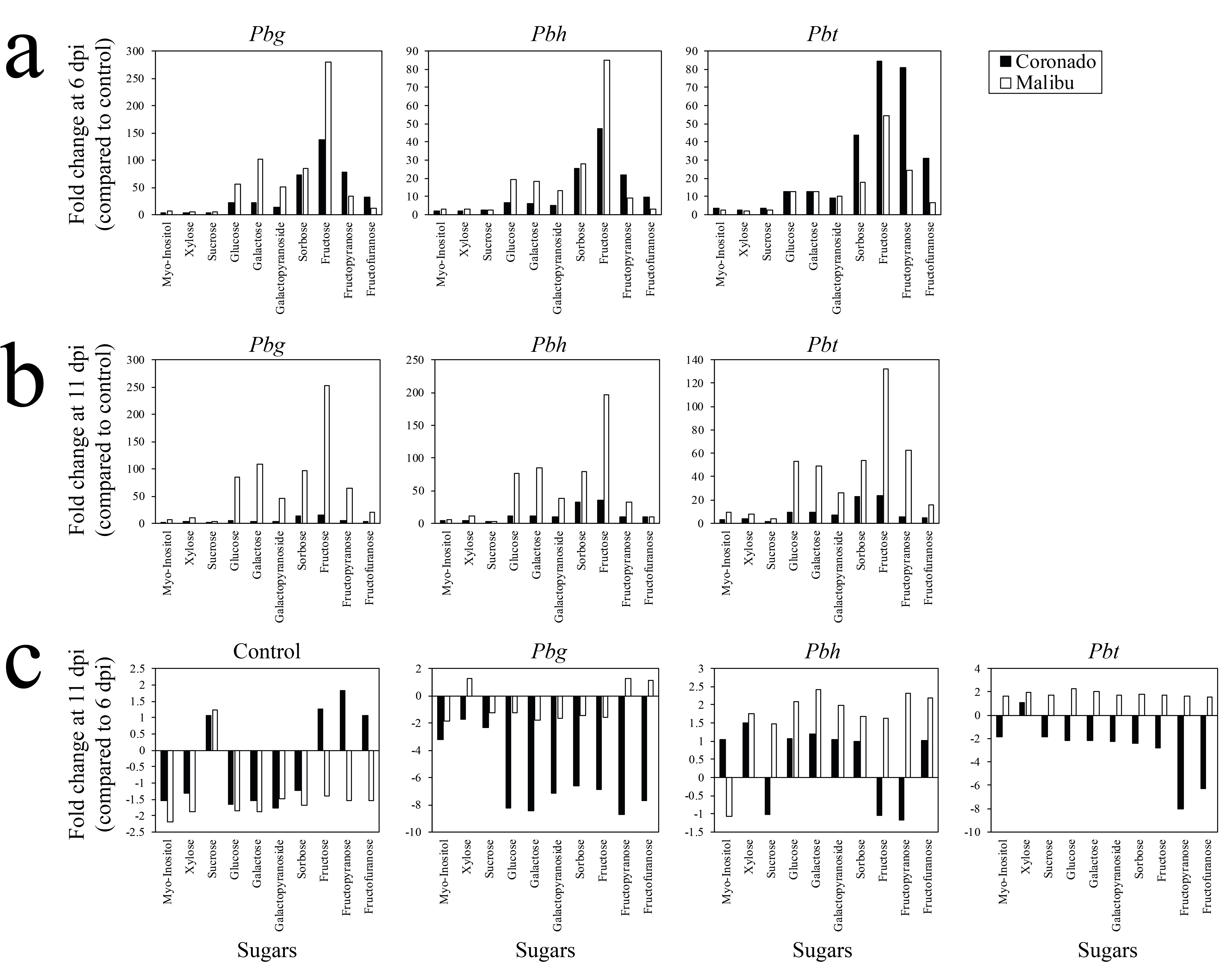


Supplementary Figure S4. *Paraburkholderia*-mediated fold change increase of soluble sugar in two Broccoli cultivars at 6 dpi (**a**), 11 dpi (**b**), and magnitude of fold change at 11 dpi compared to 6 dpi (**c**). In (**a**) and (**b**), the fold changes of treatments were calculate to non-treated control. *Pbg*: *Paraburkholderia graminis*, *Pbh*: *P. hospita*, and *Pbt*: *P. terricola*.
