# Supplementary material for: Impact of root-associated strains of three Paraburkholderia species on primary and secondary metabolism of Brassica oleracea
Source: Sci Rep. 2021 Feb 2;11:2781. doi: 10.1038/s41598-021-82238-9 (PMC7854645; doi:10.1038/s41598-021-82238-9)
Supplement: Supplementary file 3 — Supplementary Information 3. [file 41598_2021_82238_MOESM3_ESM.docx]

Impact of root-associated *Paraburkholderia* species on primary and secondary metabolism of *Brassica oleracea*

Je-Seung Jeon^1,2^, Natalia Carreno-Quintero^1,3^, Henriëtte D.L.M. van Eekelen^4^, Ric C.H. De Vos^4^, Jos M. Raaijmakers^1,2^ and Desalegn W. Etalo^1*^

^1^Netherlands Institute of Ecology NIOO-KNAW, Department of Microbial Ecology, Wageningen, 6708 PB, Netherlands. ^2^Institute of Biology, Leiden University, Leiden 2333 BE, The Netherlands; ^3^KeyGene, Wageningen, 6708PW, The Netherlands; ^4^Wageningen Plant Research, Bioscience, Wageningen, 6708 PB, The Netherlands

| **Table S1.** Analysis of variance ANOVA (type II) of shoot and root biomass percent changes in two Broccoli cultivars inoculated with three *Paraburkholderia* genera at 11 dpi. | | | | | | |
| --- | --- | --- | --- | --- | --- | --- |
| Sample | Factor | Sum Sq | Df | F value | Pr(>F) |  |
| Shoot fresh biomass relative change | Broccoli cultivars | 1425 | 1 | 4.882 | 0.040333 | * |
|  | Bacteria species | 12949 | 2 | 22.182 | 1.39E-05 | *** |
|  | Broccoli cultivars: Bacteria species | 8561 | 2 | 14.665 | 0.000166 | *** |
|  | Residuals | 5254 | 18 |  |  |  |
| Root fresh biomass relative change | Broccoli cultivars | 1210.18 | 2 | 19.711 | 3.42E-06 | *** |
|  | Bacteria species | 142.42 | 1 | 4.6394 | 0.039404 | * |
|  | Broccoli cultivars: Bacteria species | 400.55 | 2 | 6.5241 | 0.004441 | ** |
|  | Residuals | 920.94 | 30 |  |  |  |
| Signif. codes: 0 =***, 0.001 = **, 0.01 = * | | | | | | |

| **Table S2.** Analysis of variance ANOVA (type II) of root colonization assay in two Broccoli cultivars inoculated with three *Paraburkholderia* genera at two time points (6 dpi, 11 dpi). The data transformation was performed using log transformation using the package MASS in R. | | | | | | |
| --- | --- | --- | --- | --- | --- | --- |
| dpi | Factor | Sum Sq | Df | F value | Pr(>F) |  |
| 6 dpi | Broccoli cultivars | 0.0026 | 1 | 0.2463 | 0.6287 |  |
|  | Bacteria species | 8.6928 | 2 | 406.9629 | 9.41E-12 | *** |
|  | Broccoli cultivar: Bacteria species | 3.1723 | 2 | 148.5159 | 3.43E-09 | *** |
|  | Residuals | 0.1282 | 12 |  |  |  |
| 11 dpi | Broccoli cultivars | 5.7881 | 2 | 2746.36 | < 2.2e-16 | *** |
|  | Bacteria species | 1.0584 | 1 | 1004.42 | 6.14E-13 | *** |
|  | Broccoli cultivar: Bacteria species | 2.1068 | 2 | 999.65 | 4.51E-14 | *** |
|  | Residuals | 0.0126 | 12 |  |  |  |
| Signif. codes: 0 =***, 0.001 = **, 0.01 = *   \| **Table S3.** Bacteria population density of three *Paraburkholderia* species on root of two Broccoli cultivars.  *Pbg*: *Paraburkholderia graminis*, *Pbh*: *P. hospita*, and *Pbt*: *P. terricola*. \| \| \| \| \| --- \| --- \| --- \| --- \| \| Broccoli cultivar \| Rhizobacteria \| Bacterial cell number \| \| \| 6 dpi \| 11 dpi \| \| Coronado \| *Paraburkholderia graminis* \| 2.05 ± 0.11 x 10^8^ Cfu/mg roots \| 8.1 ± 0.28 x 10^7^ \| \|  \| *P. hospita* \| 1.18 ± 0.09 x 10^6^ \| 2.06 ± 0.08 x 10^7^ \| \|  \| *P. terricola* \| 1.58 ± 0.03 x 10^7^ \| 1.95 ± 0.04 x 10^7^ \| \| Malibu \| *P. graminis* \| 1 ± 0.05 x 10^8^ \| 1.02 ± 0.07 x 10^8^ \| \|  \| *P. hospita* \| 1.4 ± 0.15 x 10^7^ \| 1.53 ± 0.06 x 10^7^ \| \|  \| *P. terricola* \| 1.72 ± 0.16 x 10^6^ \| 7.02 ± 0.35 x 10^5^ \| \| Cfu: Colony forming unit *Values represent the average of 3 replicates ± SE of three replicates \| \| \| \|   Signif. codes: 0 =***, 0.001 = **, 0.01 = * | | | | | | |
|  | | | | | | |

| **Table S4**. Beta regression of disease severity index of *Xanthomonas* treated Broccoli cultivars (Coronado and Malibu). Different species of Paraburkholderia were used to prime induced systemic resistance | | | | | |
| --- | --- | --- | --- | --- | --- |
|  | model term | df1 | df2 | F.ratio | p.value |
| *Xca* | Rhizobacteria | 3 | Inf | 11.74 | <.0001 |
|  | Broccoli cultivar | 1 | Inf | 64.893 | <.0001 |
|  | Rhizobacteria:Broccoli cultivar | 3 | Inf | 20.627 | <.0001 |
| *Xcc* | Rhizobacteria | 3 | Inf | 3.811 | 0.0096 |
|  | Broccoli cultivar | 1 | Inf | 49.535 | <.0001 |
|  | Rhizobacteria:Broccoli cultivar | 3 | Inf | 7.604 | <.0001 |
